# Rehabilitation and Improvement of the Postural Function

**DOI:** 10.1155/2015/703679

**Published:** 2015-11-12

**Authors:** Thierry Paillard, Massimiliano Pau, Frédéric Noé, Luis-Millán González

**Affiliations:** ^1^Physical Activity, Performance and Health Laboratory, University of Pau and Pays de l'Adour, Department STAPS, ZA Bastillac Sud, 65 000 Tarbes, France; ^2^Biomechanics and Industrial Ergonomics Laboratory, Department of Mechanical, Chemical and Materials Engineering (DIMCM), University of Cagliari, Piazza d'Armi, 09123 Cagliari, Italy; ^3^Department of Physical Education and Sports, University of Valencia, C/Gascó Oliag 3, 46010 Valencia, Spain

Posture refers to the position of different body segments at a given time which can be modified through joint mobilization and the action of the neuromuscular system. Maintaining balance during bipedal quiet stance requires complex mechanisms from the postural control system in order to keep the vertical projection of the centre of mass (COM) within the base of support [1]. To achieve this aim, the centre of pressure (COP) plays a crucial role to compensate for any deviations of the COM, which can generate imbalance if they move beyond the limits of the base of support. The ability to control the COM depends on internal body representation in space. Internal representation is acquired by means of a learning process but also depends upon genetic factors [2]. This representation is elaborated by sensory inputs and is based on kinematic (segmental organization, whole body acceleration, and body orientation relative to earth gravity) and kinetic (joint torques and forces efforts between the plantar cutaneous surface and the ground) parameters [3]. Moreover, a postural attitude is never acquired definitively even in quiet stance. The body constantly undergoes changes caused by liquid movements and cardiac and respiratory muscular contractions. This phenomenon modifies its at-rest state and prevents it from maintaining a strict balance [4]. It is characterized by continuous body sway and results from an internal perturbation. In addition, muscle tone constantly varies which both accentuates body sway and complicates the possibility of cushioning it [5]. Postural control is thus a permanent process of balance regulation whose implementation is based on subtle mechanisms.

Postural regulation is organized in hierarchical and stereotypic patterns and requires the central integration of afferent inputs from the sensory systems as well as the motor command of antigravity muscles. The proprioceptive (myotendinous and joint sensors), exteroceptive (mainly visual and cutaneous plantar sensors, but also auditory sensors), and vestibular (vestibular sensors) inputs are integrated by the vestibular nuclei located in the brain stem and are controlled by the cerebral cortex and cerebellum [6–10]. The activation of postural muscles is organized in synergies (activation/inhibition of agonists/antagonists muscles) and is based on postural neural networks [3].

Each sensory, central, and motor component of the postural function is either healthy or pathological and will display normal or abnormal functions. In pathological subjects, the dysfunction of certain organs involved in postural control is likely to amplify body sway and/or to affect the ability to cushion it and it may also alter the segmental organization of postural control. Different evaluation methods enable the exploration of each component through protocols of motor perturbation (mechanical disturbance), sensory stimulation (sensory manipulation), and/or cognitive disturbance (e.g., virtual simulation, dual task). Postural behavior of healthy subjects can be characterized in terms of postural performance (i.e., the ability to minimize body sway) and segmental (i.e., the multijoint coordination) and neural strategies (i.e., the preferential involvement of short or long neuronal loops, i.e., myotatic or visuovestibular). A particular postural behavior can be easily considered as normal or abnormal through measures of magnitude, velocity, and acceleration of linear or angular displacements of the COM, COP, and body segments and also through measures of electromyographic activities and evaluations of the contribution of different sensory information. All these measures contribute to describe precisely the compensatory and anticipatory postural adjustments characterizing postural behavior. Compensatory postural adjustments act in a feedback manner to preserve balance in response to the actual balance disturbances whereas anticipatory postural adjustments precede the onset of a postural disturbance while minimizing its feedforward effects.

Neurological and muscular pathologies, sensitivity deficits (e.g., vestibular, visual), cerebellar syndrome, and many other diseases severely degrade postural control. The postural performance and strategy of healthy subjects notably differ from those of pathological subjects (e.g., [3, 11]). For a given pathology, the postural behaviour evolves in a specific way [12]. However, many scientific considerations in discovering testing and rehabilitation for each pathology of postural function still remain. When subjects present such pathologies, as mentioned above, they are liable to fall, which can have dramatic consequences for their physical integrity. The development of stimulation techniques of the sensory and motor functions in a rehabilitation context is likely to improve and help restore postural function while the refinement of testing techniques improves descriptions of dysfunction of the postural function. For these reasons, this special issue provides supplemental knowledge related to the evaluation and rehabilitation of the postural function in pathological subjects (from children to aged patients) that could advance their therapeutic management.

Moreover, among healthy subjects, the postural function can positively and negatively evolve according to age (e.g., development in children, involution in aged subjects) and the status of subjects in terms of physical activity (e.g., active or inactive). For all these populations, postural control can also be positively influenced by repeated regularly exercise or training. Exercise optimises the sensory, central, and motor outputs of the postural function and can induce motor program acquisitions which include specific postural adaptations [13, 14]. Indeed, in a working, leisure, or sporting context, highly skilled subjects are subjected to having a performant postural control since there is a close relationship between postural and motor skill (or postural and motor performance), specific training developing specific postural skills [15]. Postural strategy can also be modified by the effects of training [16]. The progress in the advancement of scientific knowledge in healthy subjects can help in the understanding of pathological postural mechanisms. Thus, this special issue also integrates work dealing with the effects of domestic and leisure physical activities and sport on the postural function in healthy subjects.

*Thierry Paillard* *Massimiliano Pau* *Frédéric Noé* *Luis-Millán González*
